# The Role of Resting State Networks in Focal Neocortical Seizures

**DOI:** 10.1371/journal.pone.0107401

**Published:** 2014-09-23

**Authors:** S. Kathleen Bandt, David T. Bundy, Ammar H. Hawasli, Kareem W. Ayoub, Mohit Sharma, Carl D. Hacker, Mrinal Pahwa, Eric C. Leuthardt

**Affiliations:** 1 Department of Neurological Surgery, Washington University School of Medicine, St. Louis, Missouri, United States of America; 2 Department of Biomedical Engineering, Washington University, St. Louis, Missouri, United States of America; 3 Washington University School of Medicine, St. Louis, Missouri, United States of America; 4 Department of Biomedical Engineering, Oxford University, Oxford, United Kingdom; 5 Center for Innovation in Neuroscience and Technology, Washington University School of Medicine, St. Louis, Missouri, United States of America; Indiana University, United States of America

## Abstract

**Objective:**

The role of resting state functional networks in epilepsy is incompletely understood. While some pathologic diagnoses have been shown to have maintained but altered resting state connectivity, others have implicated resting state connectivity in disease progression. However little is known about how these resting state networks influence the behavior of a focal neocortical seizure.

**Methods:**

Using data taken from invasively monitored patients with intractable focal neocortical epilepsy, we evaluated network connectivity (as determined by oscillatory covariance of the slow cortical potential (<0.5 Hz)) as it relates to neocortical seizure foci both in the interictal and ictal states.

**Results:**

Similar to what has been shown in the past for sleep and anesthesia, electophysiologic resting state networks that are defined by this slow cortical potential covariance maintain their topographic correlation structure throughout an ictal event. Moreover, in the context of focal epilepsy in which the seizure has a specific site of onset, seizure propagation is not chaotic or random. Rather, the seizure (reflected by an elevation of high frequency power) preferentially propagates along the network that contains the seizure onset zone.

**Significance:**

Taken together, these findings further undergird the fundamental role of resting state networks, provide novel insights into the network-influenced behavior of seizures, and potentially identify additional targets for surgical disconnection including informing the location for the completion of multiple subpial transections (MSPTs).

## Introduction

The brain is becoming increasingly understood as a dynamically interconnected compilation of functional networks. These networks possess both resting state and active state profiles which can be recognized by a number of signal detection modalities including electrocorticography (ECoG) and functional magnetic resonance imaging (fMRI) [1]–[8]. These networks remain intact and identifiable through both sleep and general anesthesia [7], [9]. In addition to these networks defining some of the fundamental organization of the healthy brain, understanding perturbations of this dynamic connectivity in disease states is necessary for appreciating the role these networks play in pathology [10]–[14]. As an example, beyond the normal age related alterations seen in resting state network topology [15], the default mode network (DMN) has been specifically implicated in the progression of Alzheimer's Disease (AD) with the finding that the accumulation of Aβ plaques along its circuitry contributes to aberrant DMN connectivity, likely representing a “pre-clinical” state of AD [16]. Given that the deposition of Aβ has been implicated in the disease progression of AD [17], this provides a provocative link between the structural disease process of AD and the underlying functional connectivity related to its manifestation. While these findings support the notion that the DMN in some way chronically guides the pathologic process over years, little is currently known about how a resting state network impacts a transient electrophysiologic perturbation such as a focal seizure.

There are reasons to suspect a relationship between resting state networks and epilepsy. First, pyramidal cells are the majority of cells in the neocortex [18]. These cells have a high density of dendtritic spines that project both out of cortex and locally within it. It is the distal apical dendrites that form non-local synapses that are thought to vary in their excitability and thus facilitate communication between distant regions by having matched time scales. He et al. and others have postulated that this is the cellular underpinnings of resting state networks that are either located by the negative shifts associated with slow cortical potentials or resting state networks (both of which have well correlated topographies between the modalities) [4], [9], [19]. While apical dendrites have been implicated in resting state functional networks in normal subjects they have also been implicated independently in epilepsy. In CA1 hippocampal neurons there is evidence that current in T-type calcium channels is increased specifically in apical dendrites. The hypothesis is that this phenomenon creates a situation in which fast sodium spikes in the soma back-propagate into the dendrites, whereby they detonate bursting [20]. Taken together, we would postulate that if these apical dendrites are involved in long-range connections and their variable excitability is guiding the flow of communication between regions, that in the pathological scenario of epilepsy in which apical dendrites are also implicated in seizure propagation, then this existing normal communication structure would preferentially influence the direction of pathologic activity since they may be using the same cellular substrate. Thus, seizures may, in essence, coopt the existing functional architecture.

To better understand this relationship between resting state networks and dynamic pathologic processes, we studied patients with intractable neocortical epilepsy requiring intracranial electrocorticographic (ECoG) recordings. Distinct from the structural disease process associated with AD, investigating epilepsy provides a unique model of how network connectivity is impacted by an acute event such as a seizure while also providing insight into how those networks influence the evolution of that pathologic process. This is due to the high level of both spatial and temporal resolution available through invasive recordings. The one-centimeter spatial distribution of the electrode arrays placed on the surface of the brain allows for adequate assessment of the spatial organization of network topographies. Moreover, the broad frequency scale accessible through local field potentials enables one to monitor multiple distinct physiologies. Specifically, the infraslow rhythms of less than 0.5 Hz, known as slow cortical potentials (SCPs) directly correlate with the resting state networks identified with functional MRI and the higher frequency rhythms, greater than 70 Hz, described as “fast ripples” have been associated with seizure propagation [21]. Given the durability of the SCP networks through sleep and anesthesia, we hypothesized that resting state networks would also persist through an ictal event [7], [9]. We further hypothesized that, because the negative shift of these SCPs has been previously postulated to reflect the slow rhythmic depolarization of apical dendrites associated with endogenous fluctuations in cortical excitability [22], the SCP networks would influence the propagation patterns of the seizure across cortex, if this were indeed the case. In this study we demonstrate that resting state networks are indeed persistent through a seizure facilitate the direction this pathologic activity moves through cortex. Taken together, these findings add important insights into the networked nature of seizure-related cortical pathophysiology and could provide added targets for clinical intervention.

## Materials and Methods

### Ethics Statement

The study protocol was approved by the Human Research and Protection Organization at Washington University School of Medicine. All patients provided written informed consent for study participation.

### Subjects

**Figure 1:**
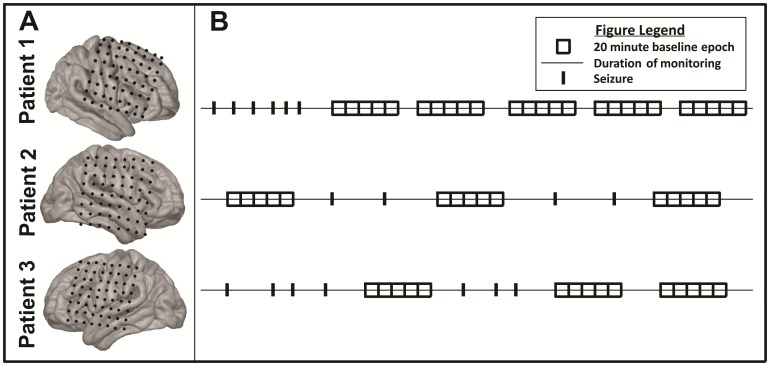
Patient Data. (A) Patient specific electrode grid coverage. (B) Graphical representation of ECoG interictal and ictal epochs selected for analysis.

Three patients undergoing invasive monitoring for characterization of their seizure foci were included in this study. Exclusion criteria included the absence of drug-resistant, focal neocortical epilepsy [23], [24]. See Table 1 for demographic and clinical information. Each patient underwent craniotomy for the implantation of subdural electrodes for invasive monitoring of their seizure focus. See Figure 1A for electrode grid coverage. Following characterization of their seizure focus with invasive monitoring, patients were taken back to the operating room for removal of their subdural electrodes and resection of their seizure focus in the usual fashion.

**Table 1:** Patient Demographics & Clinical Information.

Patient	Age	Gender	Handedness	Seizure Focus Location	Pathology	Duration of Epilepsy
1	28	F	Right	R Frontal	Normal	16 years
2	45	M	Right	R Fronto-temporoparietal	MCD	12 years
3	49	M	Right	L Fronto-temporoparietal	Non-specific gliosis	3 years

M = male; F = female; L = left; R = right; MCD = malformation of cortical development.

### Data Collection

Following a period of 24–48 hours for immediate post-surgical recovery in the intensive care unit, patients were transferred to the Epilepsy Monitoring Unit for seizure monitoring. All anti-epileptic medications were discontinued during this period. Intracranial electrode density and locations were chosen solely based on clinical indication. Physiologic recording in combination with video monitoring ensued uninterrupted for a period of 5–7 days to allow optimal characterization of patients' seizure foci.

ECoG signals were acquired from the implanted electrode array (Ad-Tech Medical Instrument Corporation, Racine, WI, USA) at a sampling frequency of 512 Hz using a differential amplifier (Natus Medical Incorporated, San Carlos, CA, USA). This amplifier was equipped with a 0.5 Hz hardware filter for clinical recording purposes. Prior to analysis, a preliminary assessment of these clinically acquired signals was performed to compare them to signals obtained via an amplifier used exclusively for research purposes (g.tec, Graz, Austria) at a sampling frequency of 1200 Hz that did not include any hardware or software filters. The covariance patterns were similar between the two data sources (see Figure S1) therefore the clinical system data was selected for further analysis because the seizure epochs were only captured on this system. Signals were referenced to skull facing ground and reference electrodes. The implanted electrodes were 2.3 mm diameter platinum electrodes embedded in silicone in an 8×8 grid orientation spaced 10 mm apart. Data were stored on a Dell PC running NicVue software (Natus-Nicolet, Middleton, WI, USA) for clinical analysis.

### Epoch Selection

Twenty minute time epochs were selected for analysis and were identified as either interictal or ictal epochs. See Figure 1B for a graphical representation of this epoch selection process. Across all patients, a total of 13 clinical seizures were identified by an epileptologist through standard clinical interpretation of the raw signals. These seizures were all in keeping with the patients' typical seizure semiology. Using a data driven method to further define seizure activity as high gamma (70–110 Hz) power activity reaching a threshold of three orders of magnitude greater than the geometric mean (as determined by a log-based scale), 4 additional subclinical seizures were identified. Ictal epochs were 20 minute windows time-locked and centered around the seizure onset. Interictal epochs were selected as 20 minute windows during which the patient did not demonstrate seizure activity and which was removed from seizure activity by at least 60 minutes on either side of recording. Between all patients, a total of 17 ictal epochs and 55 interictal epochs were identified that met these criteria for analysis.

### Network Seed Selection

The seizure focus electrode was selected as the seed for the “seizure network.” This electrode was defined by the treating neurologist as the electrode first to demonstrate ictal discharges that ultimately manifest into seizure activity. When multiple contiguous electrodes were identified as equally contributing to seizure onset, a central electrode within the seizure focus mass was selected as the seed for further network analysis. For purposes of uniform comparison, the electrode found to be most negatively correlated with the seizure onset electrode over the entire 20 minute epoch was selected for the seed electrode in the “anti-correlated network”. Simultaneous analysis of this and the epilepsy network serves as a negative control within the same dataset. Networked interactions between these two seed electrodes and all other electrodes were then completed as described below.

### Anatomic Localization & Surface Representation of Data

The electrodes were co-registered between a pre-operatively acquired T1 MPRAGE anatomic MRI sequence and a post-operatively acquired computed tomography (CT) scan as described by Hermes, et al. [25]. Correction for post-operative brain shift as identified on CT scan was made by projecting electrode coordinates to the surface of the brain along a path normal to the grid surface. Surface pial anatomy was extracted using Freesurfer 5. As previously described by Roland, et al. [26], we estimate the total amount of error introduced by our localization technique to be 2–3 mm. Following this localization, ECoG correlation and power activation maps were generated for each patient and each epoch independently. These maps were created using a 6 mm spherical Gaussian kernel centered on each electrode and then interpolated and projected onto the surface of a template brain.

### Pre-processing for Signal Analysis

Signals from every electrode were visually inspected and those electrodes identified as having poor signal to noise characteristics (amplitude greater than 10x that of the majority of electrodes in the array) were excluded from further analysis (23 of 192 electrodes, 11.9%). Remaining signals were re-referenced to the common mean for further analysis.

### Covariance Analysis

**Figure 2:**
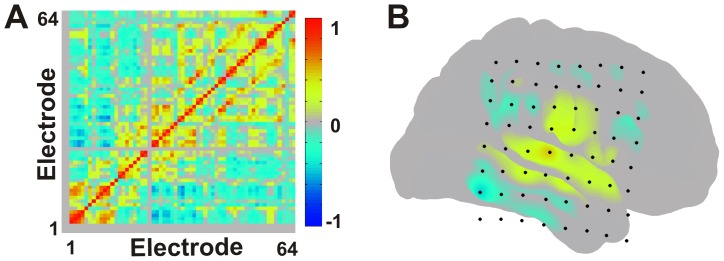
Correlation analysis. (A) Adjacency matrix depicting correlation coefficients between all electrodes across an entire 20 minute epoch (B) Cortical surface rendering of seizure network correlation structure when selecting the seizure focus seed for a single patient (Patient 2). Multiple comparison correction was performed using the false discovery rate method.

Networked interactions were evaluated as depicted in Figure 2. Re-referenced signals from the non-noisy electrodes (see above) were low-pass filtered using a 3^rd^ order digital low-pass Butterworth filter (cutoff = 0.5 Hz) to obtain the SCP. The 20 minute blocks were analyzed as single time epochs across patients. Correlation of the SCP over the 20 minute epochs was then calculated for all electrode pairs over the entire 20 minute epoch. This was completed in the following manner: The covariance between each electrode pair was calculated using Equation 1.
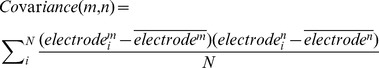
(1)


Here, 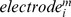 and 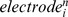 represent the *i* ^th^ of N values of the SCP signal from the *m* ^th^ and *n* ^th^ electrodes, respectively. The correlation coefficient between each electrode pair was then obtained by Equation 2:
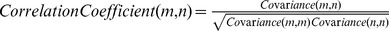
(2)


Here, electrode ‘*m*’ represents the seed electrode for a given network and it is compared against all other ‘*n*’ electrodes. Network connectivity was defined as all electrodes found to be positively statistically significantly covariant with the seed electrode as determined by a two-sample KS test of correlation coefficients (Equation 2, p value<0.05). This identified the resting state covariant networks related to both the seizure focus electrode and anti-correlated electrode seeds. The false discovery rate method was used to correct for multiple comparisons [27].

### Comparison of Spatial Correlation by Network

**Figure 3:**
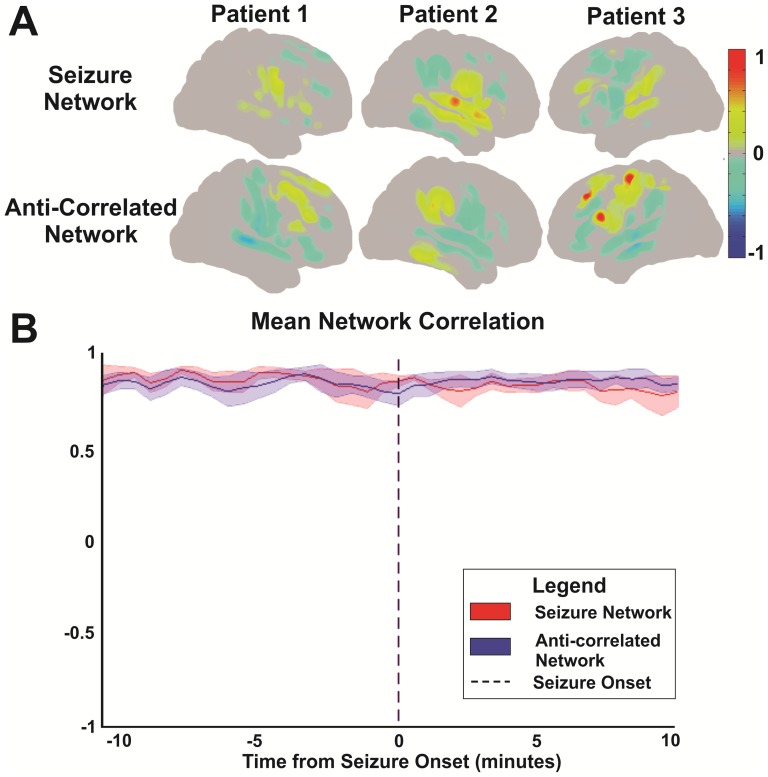
Network correlation structures. (A) Cortical surface topographies across patients for both the seizure and the anti-correlated networks. (B) SCP correlation topography over time. This plot demonstrates the spatial correlation between each network's seed-based SCP correlation map computed at 30 second intervals over the course of a 20 minute ictal epoch and that computed over the same entire 20-minute ictal epoch. This plot represents a cumulative average of spatial correlation values for all patients and all seizures. Error bars represent 95% confidence intervals across the mean as determined by a one-sample t-test. Seizure onset was time-locked to minute 10 within the 20 minute epoch.

After resting state covariant networks were defined for both the seizure focus and anti-correlated electrode seeds respectively, the topographic correlation structure for each network was then compared across time to determine network stability. This was completed by calculating the spatial correlation coeffcient between each network's correlation map computed at 30 second intervals over a single 20 minute ictal epoch and then comparing each of these to the spatial correlation structure for the entire 20 minute ictal epoch. This was done for all patients and all seizures and results are graphically depicted in Figure 3B. Error bars represent 95% confidence intervals across the mean as calculated by a one-sample t-test.

### Overall Power Analysis by Network

**Figure 4:**
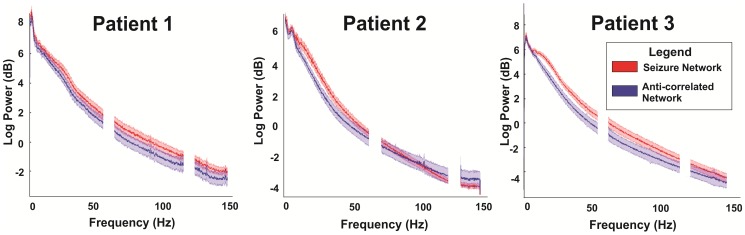
Patient specific log-normalized power spectral densities between the seizure and anti-correlated networks during an ictal epoch. Error bars represent 95% confidence intervals across the mean as determined by a one-sample t-test.

Global network power analysis was performed over an entire 20 minute epoch using a 10 second sliding window with 50% overlap via Welch's Method [28]. Power was computed for frequencies ranging from 0.5 to 150 Hz. Known noise bands at 60 and 120 Hz were avoided by band-stop filtering 30 Hz bands centered on each known noise peak. Analysis was cut off at 150 Hz as determined by the signal's noise floor. Analysis of the distribution of and variance in absolute band power followed for comparison between the subdivided “epilepsy” and “anti-correlated” network signals for both interictal and ictal epochs as shown in Figure 4. The absolute band power was log normalized to allow for comparison of the mean power distributions across networks. A one-sample t-test was then used to calculate 95% confidence intervals across the means to define significance between the two networks mean power distributions.

### Temporal Power Analysis Relative to Ictal Onset

**Figure 5:**
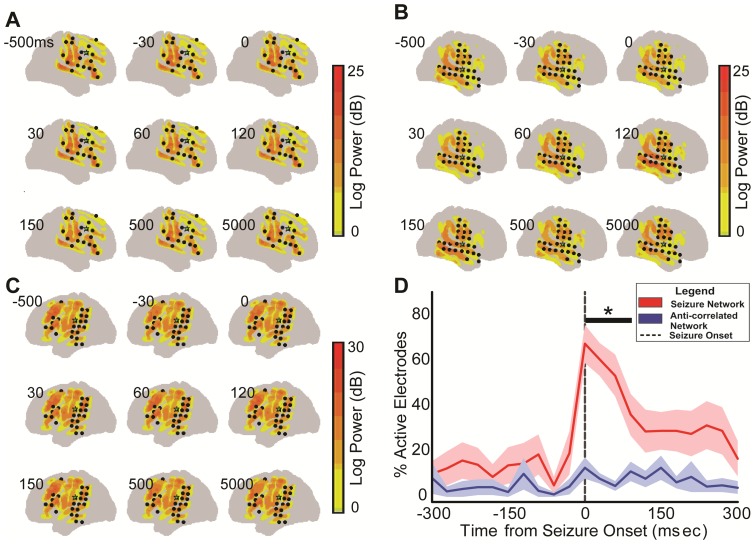
Seizure propagation analysis. Color-coded exemplar demonstrating power activations in the high gamma frequency band (70–110 Hz) for patient 1 (A), 2 (B) and 3 (C) superimposed on the cortical surface. Time is relative to seizure onset as identified by ECoG. Black dots are electrodes identified in the patient's resting state seizure network. Green star represents seizure focus electrode. (D) Averaged fractional representation of all active electrodes between the seizure and anti-correlated networks relative to seizure onset. Error bars represent standard error. *p<0.05, *post hoc* Mann-Whitney Test vs. anti-correlated network.

To determine the role of these networks as they relate to seizure propagation, the spatiotemporal dynamics of high frequency oscillations (HFOs) were also evaluated [29]. Dynamic power analysis was performed using the Fast Fourier Transform (FFT) method [30] for improved temporal resolution when compared to the global power analysis completed as above. A 30 ms sliding window with 50% overlap was used to assess high gamma frequency (70–110 Hz) power modulation over time during an entire 20 minute epoch. This frequency was chosen for two reasons. The first of which is that it is the sub-band classically seen in HFOs, while still avoiding 60 Hz noise harmonics [31]. Second, the high gamma frequency band has been shown to be associated with local cortical activations [32], [33]. The onset of ictal power activations for each electrode was defined as the first time window in which a respective electrode demonstrated power activity reaching a threshold of three orders of magnitude greater than the geometric mean as determined by a log-based scale. Mean power was determined over the first 5 minutes of a 20 minute ictal epoch so as to not include the power amplifications seen with the onset of seizure activity which was time-locked to the 10^th^ minute of the 20 minute epoch. The propagation patterns were then analyzed by identifying all active electrodes at 30 msec intervals immediately surrounding seizure onset, specifically between 500 msec before and 5000 msec after seizure onset. Active electrodes were then classified according to their network designation. Electrodes that were not included in either the seizure or anti-correlated networks were not assigned a network but were included in the total number of active electrodes. Active electrodes by network were then calculated as a percent of all active electrodes for each 30 msec time bin. This was completed for all patients and all seizures. Percent of active electrodes versus time was evaluated using Friedman's Chi-Squared tests by *post-hoc* multi-comparison corrected Mann-Whitney U tests. Results are graphically depicted in Figure 5D. Error bars represent standard error across the mean.

### Relationship Between Clinical Interpretation & Research Findings

Each subject's clinical ECoG signal had previously been interpreted by an epileptologist for mapping of their seizure focus and seizure propogation patterns during the time of grid implantation for invasive monitoring. Seizure onset electrodes and electrodes to which the seizures immediately propagated had previously been identified via this clinical interpretation. These clinically-defined electrodes were compared against the electrodes found to be included in the resting state seizure network by covariance analysis as a percent of overlap between the two groups of electrodes as depicted in Table 2.

**Table 2:** Relationship Between Clinical ECoG Interpretation & Resting State Connectivity by ECoG Covariance Analyisis.

Patient	No. Seizure onset electrodes by Clinical ECoG	No. Seizure early spread electrodes by Clinical ECoG (prior to generalization)	Correlation between Clinical ECoG seizure activity electrodes & resting state connectivity electrodes (%)	Likelihood of Clinical ECoG-defined electrode activations identified by resting state connectivity by chance (%)
1	6	6	3 of 6, (50%)	3(1/64), (4.6%)
2	2	12	10 of 12, (83.3%)	10(1/64), (15.6%)
3	2	2	1 of 2, (50%)	1(1/64), (1.6%)

All data can be made available upon request to the corresponding author.

## Results

### Resting State Network Connectivity Related to a Neocortical Seizure Focus

The seizure focus identified from the ictal onset, when used as a seed for SCP defined networks from the interictal periods, demonstrated statistically significant connectivity with distinct regions of brain. These regions of significant correlation included both near and remote cortex (see Figure 3A). When correlation values for the epilepsy and anti-correlated networks from the interictal periods were followed through into the seizure periods over the course of a 20 minute ictal epoch, there was an overall maintenance of network connectivity relative to seizure onset within each network as shown in Figure 3B.

### Ictal Power Amplification within the Epilepsy Network

Global power in the epilepsy network was then compared against global power in the anti-correlated network during periods of interictal and ictal cortical activity. As depicted in Figure 4, consistent global power amplification was seen within the epilepsy network that was greater than the power in the anti-correlated network during periods of seizure activity. Two of three patients had a statistically significant (non-overlapping 95% confidence intervals) difference between the log-normalized power spectra seen between the seizure and anti-correlated networks and the third had a trend toward this difference but did not reach statistical significance. These differences were most notable in the beta frequency band (12–25 Hz) but also seen in alpha (8–12 Hz) and gamma (30–110 Hz) to a lesser degree. These differences were reproducible across all ictal epochs.

Studies have identified broadband phenomena in the electric potentials produced by the brain that follow a power-law scaling in these signals [34], [35]. With the performance of a task, there are activity-related fluctuations in the amplitude of a power-law process lying beneath the oscillatory fluctuations. These scale-free changes are thought to represent asynchronous changes in cortical potentials that correspond to changes in the mean population-averaged firing rate. Similar to previous reporting of these task-related changes in the amplitude of this power-law process, there is an increase in amplitude of this 1/f relationship in the epilepsy network. Both networks, however, maintain a 1/f relationship. Thus, while seizures lead to an increase in global activation they do not fundamentally change the nature of the scaling relationship.

### Seizure Propagation Dynamics Related to the Epilepsy Network

Following seizure onset, the seizure network displayed an increasing percentage of all active electrodes (electrodes demonstrating power activations ≥3 orders of magnitude above the geometric mean in the high gamma frequency band, 70–110 Hz). Conversely, the anti-correlated network did not show this same progression. Temporal power analysis revealed preferential power activations within the epilepsy network (P = 1.9×10−5, χ2(9,126)  =  37.8) when compared to the anti-correlated network (P = 0.28, χ2(9,126)  =  10.96) over time, relative to seizure onset, as shown graphically in Figure 5. *Post-hoc* comparisons showed significant differences in percent of activated electrodes between the epilepsy and anti-correlated networks continuing until 90 ms following seizure onset.

### Clinical Correlation to Research Findings & Seizure Outcomes

Table 2 defines the percent overlap between the epileptologist's interpretation of the clinical ECoG tracing and the resting state connectivity as defined by ECoG covariance analysis. The resting state seizure network captured an average of 61% of the electrodes identified to be active during the initial seizure onset and early propagation (50%, 83% and 50% for patients 1, 2 and 3, respectively). This is compared to an average expected activation correlation rate of 7.3% assuming no association between the clinical ECoG tracing and the resting state connectivity as defined by ECoG covariance analysis with an equal likelihood of resting state activation at each of the 64 electrodes (1.6% chance of activation at each electrode, (1/64)).

Table 3 summarizes the clinical seizure outcomes in the 3 subjects included in analysis. Two of the 3 subjects remain seizure free following resection of their seizure focus as identified by invasive monitoring. The remaining patient has experienced a dramatric reduction in seizure burden when compared to their preoperative seizure frequency (daily seizures pre-operatively compared to one seizure every 2 months post-operatively). Table 3 also defines the volume of tissue resected at the time of surgery for each patient. As the eloquence of cortext surrounding the seizure focus permited, the seizure onset electrodes and immediately adjacent cortex was resected to a depth of 1.5 cm to allow the maximum sulcal depth to be included in the surgical resection.

**Table 3:** Surgical Resection & Seizure Outcomes.

Patient	Resected tissue dimensions*	Pre Op Seizure Frequency	Post Op Seizure Frequency**
1	4.5 cm×3.5 cm×1.5 cm	One seizure per day	One seizure every 2 months
2	4.5 cm×3 cm×1.5 cm	One seizure every 3 weeks	Seizure free on medication
3	6.1 cm×2.1 cm×1.5 cm	Two seizures per day	Seizure free on medication

*As measured from en bloch specimen by pathologist; dimensions are length x width x depth.

**Seizure outcome determined 6 months postoperatively.

## Discussion

Using data taken from invasively monitored patients with neocortical intractable epilepsy, this study demonstrates the important relationship that exists between resting state networks and the physiology associated with a seizure. Similar to what has been shown in the past with sleep and anesthesia for electophysiologic resting state networks that are defined by slow cortical potentials [9], [36], these networks maintain their topographic correlation structure throughout the ictal event. Moreover, in the context of focal epilepsy in which the seizure has a specific site of onset, the seizure propagation is not chaotic or random. Rather, the seizure (reflected by an elevation of high frequency power) preferentially involves the network that contains the seizure onset zone. Taken together, these findings support that pathologic seizure activity topographically evolves in the anatomic distribution defined by areas that share SCP covariance with the seizure onset zone. These findings further undergird the fundamental role of resting state networks, provide novel insights into the network-influenced behavior of seizures, and potentially identify more informed targets for surgical intervention, specifically guiding the location of multiple subpial transections (MSPTs) at the time of resection.

### Physiologic Implications

In prior studies, the durable nature of resting state networks has been shown in states associated with reduced or suppressed cognitive activity, including sleep and anesthesia [9], [36], [37]. This study is the first to show that in the presence of an acutely disruptive event, such as a seizure, that this connectivity is still maintained. These findings on the surface could be seen to conflict with prior work by Pizoli, et al. [38]. In this study the authors evaluated a five-year-old boy with a protracted one-year history of severe epileptic encephalophathy who on functional MRI demonstrated reduced connectivity. Once undergoing successful corpus callosotomy surgery, subsequent serial imaging revealed a restoration of his functional networks. Here, in this work, the networks appear relatively unperturbed by a seizure. There may several reasons for the differences between these studies. First, one difference may be developmental in nature. The work by Pizoli, et al. studied a child, while our work studies adults. Thus, it could be that the seizures in a developing brain could impair resting state network formation, but once formed these networks are more durable in the presence of seizures. Also, the patients in this study had focal seizure onset that then secondarily generalized, while in Pizoli, et al.'s work the type of epilepsy studied was generalized across both hemispheres. Thus, a more generalized pathologic process may globally influence the network architecture while a focal pathology may be better accommodated.

The maintenance of resting state connectivity between seizure foci and remote cortex as determined by SCP correlation during both interictal and ictal epochs indicates that seizure foci are functionally coupled with remote, non-epileptogenic cortex at rest. This notion is not novel. The concept of epilepsy networks was first postulated by Spencer in 2002 [39], but advances in our understanding of resting state connectivity over the past decade have contributed to an appreciation of what this underlying neuronal modulation may represent. Infraslow cortical fluctuations in the resting state blood oxygen level-dependent (BOLD) signal by fMRI [4], [40]–[43] and its electrophysiologic correlate of SCPs [7] identify cortical regions that are functionally interconnected. The fact that this connectivity is identified in the setting of a neocortically based seizure focus suggests that the epileptogenic cortex subserving seizure activity is also connected with seemingly normal, remote cortical sites which have not been previously implicated in the pathogenesis of epilepsy. Therefore, in addition to the pathologic cortical focus identified to be associated with seizure onset, there is a more widely distributed region of potentially pathophysiologic connections that might be relevant to the disease process.

The pathologic nature of these connections are highlighted by the differential behavior of the seizure with anatomic sites that show significant SCP correlation with the onset zone as compared to those areas that are anti-correlated. There was relatively increased global power amplification within the “seizure network” when compared to the “anti-correlated network.” This relative amplification in overall power across frequency bands suggests several possibilities. First, because these different rhythms (i.e. mu, beta, gamma) have been associated with different underlying neural circuits [5], [44] this increase represents involvement of both thalamocortical and cortical sources [45]–[47]. Alternatively, as has been posited by Miller, et al. [34] that the power law relationship seen in cortical physiology is the summation of purely asynchronous neuronal firing in cortex. This would suggest that this ictal increase is simply a general increase in cortically based asynchronous activity. Either way, that this activity is preferentially occurring in a topography that can be identified at rest, and is maintained during the seizure, strongly supports that this pathologic physiology is not randomly distributed after its onset. Rather, it appears to be distributed over a proscribed topography defined by resting state connectivity.

Beyond a general topographic difference in pattern of abnormal seizure activity, it appears that the focal cortical activation (as measured by high gamma power) emanating from the seizure focus moves preferentially along the interictally defined resting state network. Conversely, the areas shown to be anti-correlated with the seizure onset zone did not show a similar progression. In the setting of Alzheimer's Disease, previous work by Sheline et al. has demonstrated a preferential deposition of beta-amyloid plaques along anatomic regions defined as resting state networks. The group refers to this functional network-defined ordering of structural abnormalities as a “structural-functional” connection between functional connectivity and disease progression in AD [16]. As a complement to this notion, in the setting of epilepsy where there are rapid dynamic pathologic events (distinct from the chronic progressive changes with AD), seizure propagation along the resting state networks suggests an ictal-functional connection between pathologic process of focal epilepsy and the functional architecture of the brain. Taken together, the resting state connectivity, spectral profile and propagation dynamics related to focal epilepsy suggest a network driven process that is, to a degree, a more ordered phenomenon than is typically assumed early into the secondary generalization of a focal seizure.

### Clinical Implications

Medically refractory epilepsy affects nearly one million Americans annually [48]. Surgery offers a potential benefit to approximately a third of these patients who are found to have focal epilepsy. The current clinical practice for the surgical management of medically refractory epilepsy at tertiary epilepsy centers includes pre-operative evaluation with dedicated epilepsy protocol magnetic resonance imaging (MRI), inpatient video electroencephalography (EEG), interictal fluorodeoxyglucose Positron Emission Tomography (FDG-PET) and neuropsychological evaluation. Patients who don't have strong concordance between EEG and imaging findings or who have normal imaging studies are typically referred for invasive monitoring for further characterization of their seizure foci via electrocorticography. If a non-eloquent cortical region is identified as the seizure focus, then the patient is a candidate for surgical resection of this focus with or without adjuvant multiple subpial transections (MSPTs). If part or all of the cortical region identified as the seizure focus is located within eloquent cortex (cortex subserving speech, motor function, vision, etc) then the patient is not a candidate for surgical resection but rather may undergo palliative MSPTs alone. MSPTs are shallow, orthogonally oriented cortical incisions that are in parallel with the laminar cytoarchitecture of the cortex and serve to limit seizure spread without significantly impacting the relevant cortical function [49]. MSPTs are typically made in a circumferential pattern surrounding the identified seizure focus and have not previously been associated with an informed surgical target to prevent seizure propagation. With these techniques, surgical cure rates for focal epilepsy with seizure focus resection remain 70% at 6 months and fall off to 50% by 2 years following surgery. For unresectable seizure foci with MSPTs alone the prognosis is worse [49]–[53].

Enhanced understanding of network connectivity related to seizure foci may improve this surgical cure rate by a number of mechanisms. First, for those patients found to harbor unresectable seizure foci, the seizure network may represent a more informed manner in which MSPTs can be applied over the cortical surface in and around the seizure focus. Alternatively, given the ictal propagation dynamics along the seizure network, completing MSPTs along this network may enhance seizure control even following resection of patients' seizure foci. Taken together, these findings may thus optimize clinical outcomes for focal neocortical epilepsy by providing cortical transection therapies when frank resection is not possible (i.e. offering an alternative treatment option for a clinically frustrating scenario) or after surgical resection of a seizure focus (i.e. making an effective therapy even better).

### Limitations

There are several limitations to this study that deserve attention. These include a relatively small sample size of three patients as well as limited electrode grid coverage. Each patient's cortical coverage was dictated exclusively by clinical circumstances therefore our findings should only be interpreted in the context of this limited coverage. It is certainly possible that this limited cortical coverage impacts the implications of our findings, particularly if the seizure onset focus was in fact not included in the cortical region from which ECoG was recorded. While this is possible, the relatively favorable seizure outcomes across all three patients suggest that the seizure focus was included in analysis and then subsequently resected. There are however other potential implications of this limited electrode coverage which remain, including more extensive resting state connectivity that has not been appreciated or additional physiologic phenomena that have not been recognized due to the small area of cortex sampled for this clinical purpose. A third limitation is the possibility that the relationship between resting state connectivity and seizure propagation described herein is not necessary for seizure propagation but rather coincident with seizure pathophysiology. Studies investigating a relationship of necessity versus coincidence would need to be completed in the future to investigate this further. A fourth limitation is that by understanding the resting state connectivity and targeting it with MSPTs at the time of surgery, it is possible that new connectivity will develop and produce new propagation and resting state connectivity patterns. Further studies will need to be designed to investigate this possibility as well. Finally, our findings are applicable exclusively to focal neocortical epilepsy. As such, the cortical physiologic phenomena related to other patterns of epilepsy still remain unknown.

## Conclusions

In the setting of focal neocortical epilepsy, the pathologic site of seizure onset demonstrates networked interactions with broad anatomic regions on the cortical surface at rest and during a seizure. These seizure-related networks, which are present during interictal recording, are preferentially involved with the propagation and anatomic distribution of the ictal physiologic event. These findings support a more ordered, network driven phenomenon that could have implications for the surgical management of the disease.

## Supporting Information

Figure S1 **Comparison of clinical and research covariance topographies.** Exemplar cortical surface topography for a 20 minute interictal epoch aquired for clinical purposes (A) using a Natus differential amplifier (Natus Medical Incorporated, San Carlos, CA, USA) which includes a 0.5 Hz hardware filter and a 20 minute interictal epoch acquired for research purposes (B) using a g.USBamp amplifier (g.tec, Graz, Austria) which includes no hardware or software filter.
(TIF)
