# Supplementary figures and images for: The Role of Resting State Networks in Focal Neocortical Seizures
Source: PLoS One. 2014 Sep 23;9(9):e107401. doi: 10.1371/journal.pone.0107401 (PMC4172478; doi:10.1371/journal.pone.0107401)

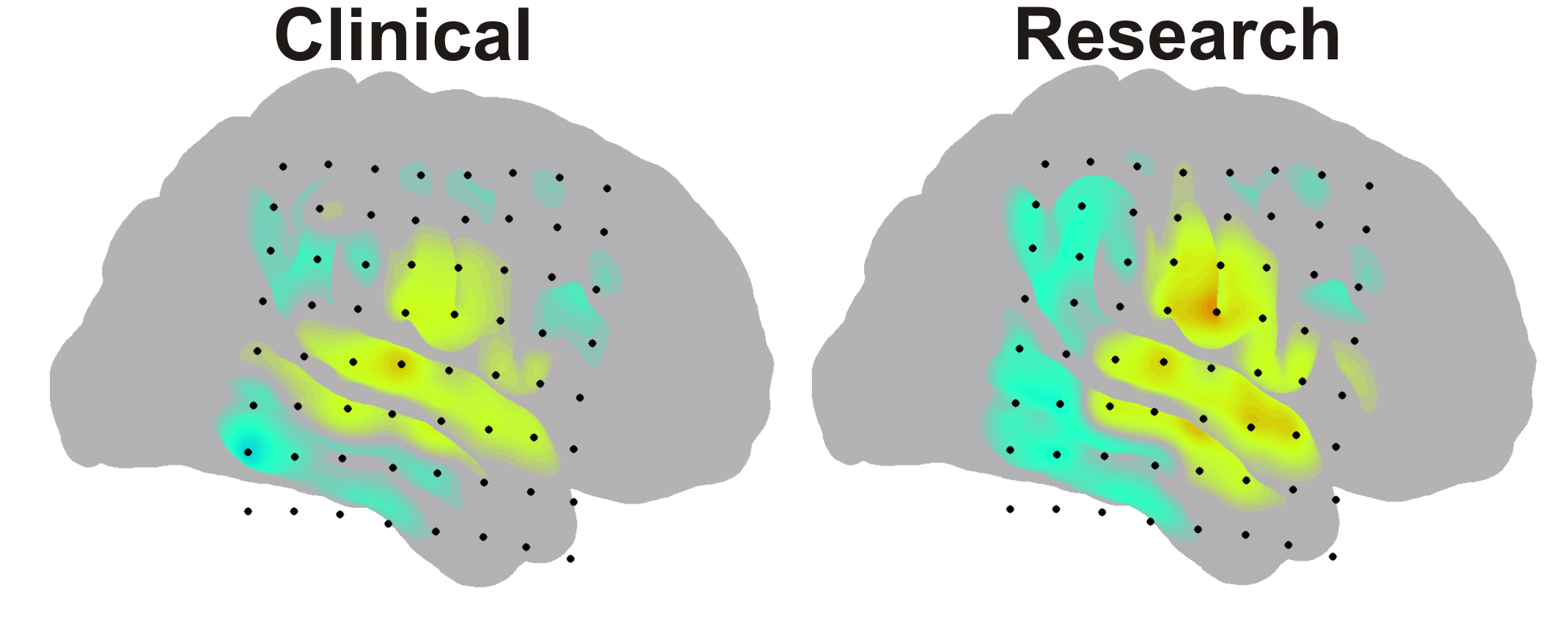

Supplement: Figure S1 — Comparison of clinical and research covariance topographies. Exemplar cortical surface topography for a 20 minute interictal epoch aquired for clinical purposes (A) using a Natus differential amplifier (Natus Medical Incorporated, San Carlos, CA, USA) which includes a 0.5 Hz hardware filter and a 20 minute interictal epoch acquired for research purposes (B) using a g.USBamp amplifier (g.tec, Graz, Austria) which includes no hardware or software filter. (TIF) [file pone.0107401.s001.tif]
